# A bibliography study of *Shewanella oneidensis* biofilm

**DOI:** 10.1093/femsec/fiad124

**Published:** 2023-10-05

**Authors:** Shan Chen, Yuanzhao Ding

**Affiliations:** Department of Applied Social Sciences, The Hong Kong Polytechnic University, 11 Yuk Choi Rd, Hung Hom, Hong Kong, China; School of Geography and the Environment, University of Oxford, South Parks Road, Oxford OX1 3QY, United Kingdom

**Keywords:** bibliography study, biofilm, extracellular polymeric substances, matrix, *Shewanella oneidensis*

## Abstract

This study employs a bibliography study method to evaluate 472 papers focused on *Shewanella oneidensis* biofilms. Biofilms, which are formed when microorganisms adhere to surfaces or interfaces, play a crucial role in various natural, engineered, and medical settings. Within biofilms, microorganisms are enclosed in extracellular polymeric substances (EPS), creating a stable working environment. This characteristic enhances the practicality of biofilm-based systems in natural bioreactors, as they are less susceptible to temperature and pH fluctuations compared to enzyme-based bioprocesses. *Shewanella oneidensis*, a nonpathogenic bacterium with the ability to transfer electrons, serves as an example of a species isolated from its environment that exhibits extensive biofilm applications. These applications, such as heavy metal removal, offer potential benefits for environmental engineering and human health. This paper presents a comprehensive examination and review of the biology and engineering aspects of *Shewanella* biofilms, providing valuable insights into their functionality.

## Introduction

Biofilms are formed when microorganisms attach to surfaces or interfaces, giving rise to populations or communities. These biofilms are composed of microorganisms that are embedded in extracellular polymeric substances (EPS), which provide a framework and protection for the microorganisms (Yang et al. 2012). EPS constitute more than 90% of the biomass in biofilms, with cells accounting for less than 10% (Flemming and Wingender 2010). Microorganisms within biofilms exhibit physiological differences compared to those in planktonic stages, demonstrating increased resistance to antibiotics and tolerance to harsh environments (Stewart and Franklin 2008). Understanding biofilms is of paramount importance due to their applications in environmental engineering, such as trickling biofilm reactors, as well as their significant role in the field of medical science, where more than 80% of human infections are associated with biofilms (Parker et al. 1989, Battistoni et al. 1992). Investigating biofilms can pave the way for the design of more efficient biofilm-based applications and the development of antibiofilm drugs for public health purposes.

The process of biofilm formation involves sequential stages of attachment, maturation, and dispersion. The attachment stage typically lasts for 4–12 h, followed by a maturation stage lasting ~12–72 h, after which the biofilms disperse (Dominguez-Zacarias et al. 2006, Takahashi et al. 2010). Surface modification research is focused on either preventing or enhancing biofilm formation (Bazaka et al. 2012, Hou et al. 2013), which is crucial in the context of preventing biofilms on medical devices as well as improving biofilm formation in biofilm reactors (Ding et al. 2014). Moreover, the interactions between biofilms and antibiotics are a significant area of investigation (Anwar et al. 1989) due to the high prevalence of biofilm-related human infections (Wagner and Iglewski 2008, Arif et al. 2010, Bjarnsholt 2013).

The biofilm matrix is primarily composed of proteins, polysaccharides, extracellular DNA (eDNA), and lipids. These components interact and assemble into three-dimensional structures that serve as the scaffold for the biofilm matrix. The interplay between these components involves various forces, including electrostatic attraction, ionic attraction, repulsion, hydrogen bonding, and van der Waals interactions (Flemming and Wingender 2010). However, several key questions pertaining to the biofilm matrix remain unanswered, such as the specific functions of each component, the primary forces governing their interactions, and the influence of metabolites on matrix stability. Gaining insights into these aspects of the biofilm matrix is of utmost importance for constructing resilient biofilms, preventing biofilm formation, enhancing biofilm-based environmental applications, and designing efficacious antibiofilm drugs.

This study provides a comprehensive analysis of 472 published articles to explore the potential of *Shewanella oneidensis*, an environmentally isolated bacterium known for its ability to reduce metals. *Shewanella oneidensis* has been extensively utilized for applications such as uranium immobilization and chromium reduction, thanks to its metal-reducing capabilities (Myers and Nealson 1988, Cao et al. 2011, Ding et al. 2014). Moreover, its nonpathogenic nature, adaptability to anaerobic and aerobic environments, and capacity to form resilient and cohesive biofilms make it highly promising for future environmental applications. In comparison to enzyme-based biocatalysis, *S. oneidensis* biofilm-based biocatalysis offers enhanced stability in natural environments, rendering it well-suited for environmental bioreactors and various applications, including the removal of heavy metals. This review primarily focuses on *S. oneidensis* as a model organism and investigates its biofilm matrix, aiming to provide valuable insights that contribute to the field of environmental engineering.

## Materials and methods

In June 2023, a comprehensive data collection was conducted using the widely recognized bibliographic database, Web of Science, which encompasses various subdatabases. This choice was made to ensure the reliability and extensive utilization of the collected data. The search profile specifically focused on “*Shewanella oneidensis* biofilm.” Web of Science was selected as the primary database due to its reputation as a trusted resource widely employed within the academic community.

To generate visual representations for the bibliographic analysis, the powerful data visualization tool, VOSviewer, was utilized. The downloaded data files were imported into VOSviewer, enabling the manipulation and adjustment of parameters based on the specific analysis objectives and the diverse data sources available. It is important to note that the creation of maps using web data often requires data cleaning processes to ensure accuracy and reliability. Therefore, VOSviewer facilitated efficient handling of such data cleaning procedures, contributing to the production of robust and meaningful visualizations.

Unless otherwise specified, the mapping conducted using VOSviewer followed the default settings as per previous studies (Meng et al. 2020, Cavalcante et al. 2021, Huang et al. 2022). In the keyword study, a minimum keyword occurrence of “20” was selected. For the country study, a minimum of “5” documents from a country were required for inclusion. Similarly, for the organization study, a minimum of “5” documents from an organization were considered for analysis.

## Results

**Figure 1.**
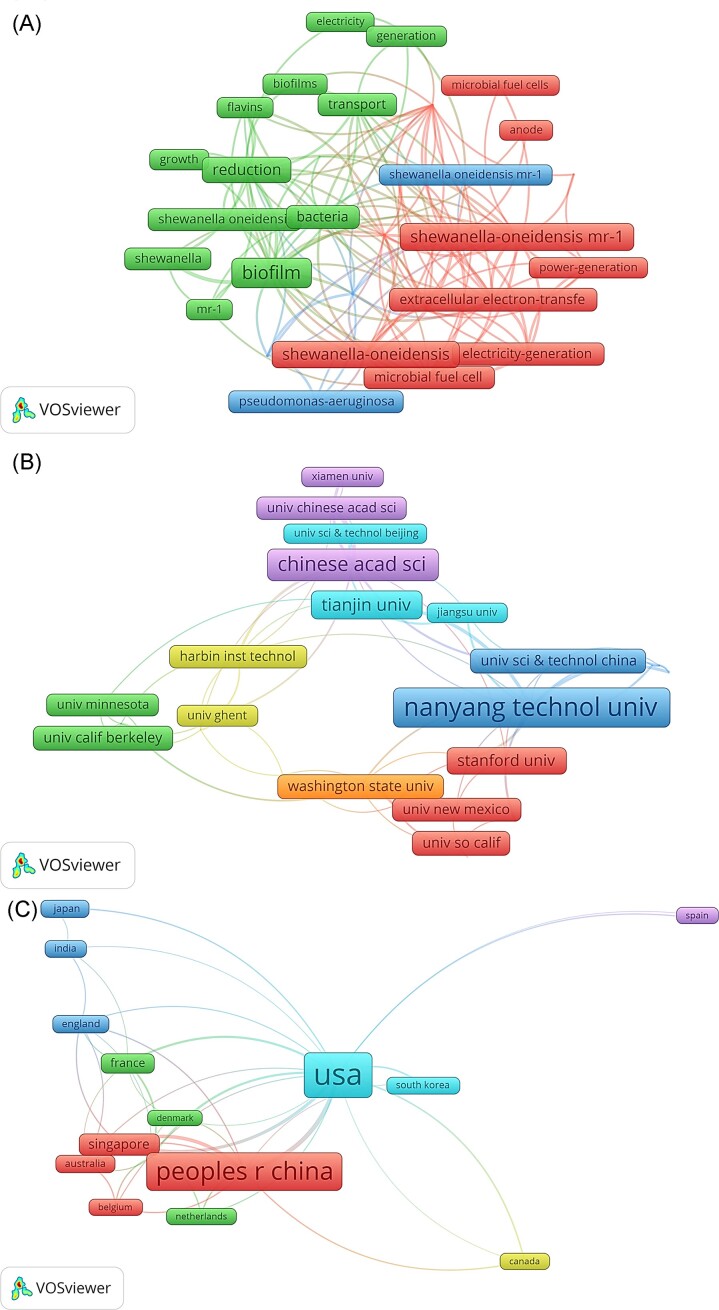
VOSviewer’s analysis of (A) keywords, (B) organizations, and (C) country/region. Fig. 1(a): keyword clusters—VOSviewer identifies thematic clusters by analyzing keyword co-occurrence. Colors denote distinct thematic groups of frequently associated keywords. Fig. 1(b) and (c): collaboration networks—colors in these networks indicate varied collaborative clusters among connected institutions or countries.

Table 1 provides a succinct overview of the recent strides made in *S. oneidensis* biofilm research. Delving into the keyword analysis presented in Fig. 1(a), a multitude of keywords are intricately linked to the process of electron transfer, encompassing terms like “extracellular electron transfer,” “electricity generation,” “microbial fuel cell,” and “power generation.” Further, the keywords intricately tied to the biofilm matrix, such as “growth,” “transport,” and “flavins,” offer an insightful glimpse into the multifaceted aspects of this intricate biological phenomenon.

**Table 1.** Significant development of *S. oneidensis* biofilm.

Main findings	Organization	Country	Reference
Pyruvate supporting the survival of *S. oneidensis* MR-1 under stationary-phase conditions.	Stanford University	USA	Meshulam-Simon et al. (2007)
Metabolic energy for maintaining cell attachment	Stanford University	USA	Saville et al. (2011)
c-di-GMP as a key intracellular regulator for controlling biofilm stability	Stanford University; Nagoya University	USA; Japan	Thormann et al. (2006)
Engineered *S. oneidensis* biofilm working efficiently in heavy metal removal	Nanyang Technological University; Temasek Life Sciences Laboratory; Agency for Science, Technology and Research	Singapore	Ding et al. (2014)
*In situ* chemical mapping of *S. oneidensis* biofilm	Nanyang Technological University; Pacific Northwest National Laboratory	Singapore; USA	Ding et al. (2014)
Toxic effect in *S. oneidensis* biofilm by chemical mapping	Nanyang Technological University; Pacific Northwest National Laboratory; Western University of Health Sciences	Singapore; USA	Ding et al. (2019)
Enhancing bidirectional electron transfer of *S. oneidensis* by a synthetic flavin pathway	Tianjin University; Nanyang Technological University	China; Singapore	Yang et al. (2015)
Methane production by acetate dismutation stimulated by *S. oneidensis* and carbon materials	Chinese Academy of Sciences; Qingdao National Laboratory for Marine Science and Technology; Aix-Marseille Univ; Kunming University of Science & Technology; Guangdong Institute of Eco-environmental Science and Technology	China; France	Xiao et al. (2020)
Selectively facilitating the electron acceptance of methanogens by riboflavin	Chinese Academy of Sciences; Institute of Eco-environmental and Soil Sciences, Guangdong Academy of Sciences; Pilot National Laboratory for Marine Science and Technology	China	Liu et al. (2022)
Molecular underpinnings of Fe(III) oxide reduction by *S. oneidensis*	Pacific Northwest National Laboratory; University of East Anglia	USA; UK	Shi et al. (2012)
Electrically conductive bacterial nanowires produced by *S. oneidensis*	Pacific Northwest National Laboratory; University of Guelph; Gwangju Institute of Science and Technology; Marine Biotechnology Institute; Pennsylvania State University; University of Southern California	USA; Canada; Korea; Japan	Gorby et al. (2006)
Influence of outer membrane c-type cytochromes on particle size and activity of extracellular nanoparticles produced by *S. oneidensis*	Nanyang Technological University; National University of Singapore; University of New South Wales	Singapore; Australia	Ng et al. (2013a)
Novel reduction of mercury(II) by mercury-sensitive dissimilatory metal-reducing bacteria	Rutgers University	USA	Wiatrowski et al. (2006)

Moving on to the organization analysis, showcased in Fig. 1(b), it becomes evident that several institutions have assumed pivotal roles in propelling *S. oneidensis* biofilm research forward. For instance, the Environmental Microbial Biofilm Biotechnology (EMBB) Group at Nanyang Technological University, Singapore, delved into the influence of outer membrane c-type cytochromes on the particle size and activity of extracellular nanoparticles produced by *S. oneidensis* (Ng et al. 2013a). The EMBB group further advanced their research by engineering *S. oneidensis* to efficiently remove the heavy metal pollutant Cr(VI) from water (Ding et al. 2014), alongside their significant contribution to the development of microbial fuel cells based on *S. oneidensis* to optimize electricity generation efficiency (Yang et al. 2015). Moreover, the research endeavors spearheaded by Alfred Spormann’s team at Stanford University explored the intricate landscape of *Shewanella*’s environmental systems biology (Fredrickson et al. 2008). This exploratory journey also encompassed a thorough investigation into the attachment and detachment processes of *S. oneidensis* (Thormann et al. 2004, 2005, 2006), shedding light on crucial aspects of its behavior. The team’s studies further ventured into the domains of hydrogen metabolism and energy-dependent stability within *S. oneidensis* (Meshulam-Simon et al. 2007, Saville et al. 2011). Notably, Liu Fanghua’s research group from the Chinese Academy of Sciences made significant strides by unraveling the potential of *S. oneidensis* in methane production (Xiao et al. 2020) and unraveling the intricate electron transfer process within *S. oneidensis* (Liu et al. 2020, 2022).

Within our comprehensive country analysis, revealed in Fig. 1(c), a constellation of nations has emerged as noteworthy contributors to the domain of *S. oneidensis* biofilm research. These nations include a diverse array of participants such as the USA, China, UK, France, Australia, Canada, Spain, the Netherlands, Belgium, Denmark, Japan, South Korea, India, and Singapore. It is particularly intriguing to observe that Western nations, including the USA, UK, France, Australia, Canada, Spain, Netherlands, Belgium, and Denmark, primarily focused their research on unraveling the intricate metabolic intricacies of *S. oneidensis* biofilms. Their efforts were channeled toward an in-depth comprehension of the underlying processes and reactions within these biofilms, offering a profound understanding of their metabolic capacities and potential applications. On the other hand, Asian nations such as China, Japan, South Korea, India, and Singapore demonstrated a marked emphasis on application-oriented studies concerning *S. oneidensis* biofilms. Their research endeavors were dedicated to exploring the practical applications and potential utilities of this biofilm across various fields. Through these explorations, they aspired to harness the potential of *S. oneidensis* as a sustainable and ecofriendly solution, with applications spanning bioremediation, renewable energy, and wastewater treatment. Notably, the collaborative efforts across international boundaries and the diversified spectrum of research initiatives have substantially enriched our comprehension of *S. oneidensis* biofilms. The fusion of expertise and perspectives from both Western and Asian nations has engendered a wealth of insights into the foundational metabolic processes and the pragmatic applications of this captivating biofilm phenomenon.

## Discussion

### Unlocking the potential of catalysis in diverse environments

Biofilms are formed by the aggregation of microorganisms at surfaces or interfaces, where they are embedded within self-produced EPS, creating a population or community. These biofilms are present ubiquitously, spanning from natural environments to human diseases (Hall-Stoodley et al. 2004). In natural settings, there are typically two distinct stages of cellular existence: the planktonic stage and the biofilm stage (Daw et al. 2012). This is primarily due to the protective nature of EPS, which serves as a “House of the biofilm cells,” safeguarding the biofilm cells within the matrix (Flemming et al. 2007).

This novel character contributes to the field of biotechnology, particularly in the development of biofilm-based catalysis. Unlike conventional biocatalysis, which primarily relies on enzyme-based catalysis(Csoregi et al. 2001, Sjode et al. 2008). this character offers a catalytic approach that is less sensitive to environmental fluctuations. Enzyme-based catalysis, while efficient, can be easily affected by small temperature changes, resulting in fluctuations in the reaction. Therefore, there is a strong demand for a biotechnology that exhibits less sensitivity in catalysis.

Biofilm-based catalysis shows promising potential in environmental applications due to the increased tolerance and stability of biofilm cells within their environment. Presently, biofilm-based trickling filter reactors have been established to enhance the catalytic reactions (Parker et al. 1989, Battistoni et al. 1992). These reactors employ biofilms to improve the efficiency and stability of the catalytic process, providing a more robust and reliable approach for environmental applications.

### Dynamics of biofilm formation: attachment, maturation, and dispersal processes

Biofilms undergo a distinct life cycle characterized by five primary stages: (1) initial attachment, (2) irreversible attachment, (3) early maturation, (4) late maturation, and (5) dispersion. The attachment stage typically spans a period of 4–48 h, and it plays a crucial role in biofilm formation (Gottenbos et al. 2000, Thormann et al. 2004, Das et al. 2010, Tian et al. 2014). During this stage, eDNA and polysaccharides perform vital functions in the initial attachment process, ultimately influencing the overall biofilm formation (Jermy 2010, Orgad et al. 2011). Surface modification techniques, including adjustments to surface hydrophobicity and other factors, can also impact the initial attachment of biofilms (Stanley 1983, Takahashi et al. 2010, Bazaka et al. 2012).

The maturation stage, which typically lasts around 48–144 h, is characterized by the development of mature biofilms exhibiting three-dimensional (3D) structures, such as mushroom-like formations, with a thickness ranging from 10 to 100 μm. Subsequently, as a response to oxygen and nutrient limitations, cells within the mature biofilms initiate the dispersion process (Dominguez-Zacarias et al. 2006, Rice et al. 2009, Ding et al. 2014). Dispersal cells are released into the surrounding environment, seeking surfaces for reattachment and initiating the initial attachment phase once again. The primary modes of motility for these cells include swimming, swarming, and twitching (Di Bonaventura et al. 2008, Pompilio et al. 2008, Kearns 2010).

### Exploring the role of matrix components in *S. oneidensis* biofilms: proteins, polysaccharides, eDNA, and lipids

In the biofilm matrix, proteins, polysaccharides, eDNA, and lipids are the main components. In terms of biomass, proteins and polysaccharides collectively account for more than 90%, with the protein-to-polysaccharide ratio typically ranging from 1:1 to 5:1 depending on the species (Yang et al. 2012). These matrix components interact with each other to form the scaffold of the biofilm matrix. The forces between these components include electrostatic attraction, ionic attraction, repulsion, hydrogen bonding, and van der Waals interactions (Flemming and Wingender 2010).

Regarding proteomics studies of *S. oneidensis*, it has been found that the genome contains a total of 4758 predicted protein-encoding open reading frames (CDSs), with ~54.4% of the proteins assigned a biological function (Heidelberg et al. 2002). Among these proteins, a large 285 kDa protein called Bap/RTX hybrid cell surface protein (SO4317) has been identified as a key mediator of biofilm formation in *S. oneidensis* MR-1. This protein, known as the biofilm-promoting factor A (BpfA), plays a crucial role, as demonstrated by the observation that knockout of the BpfA gene leads to a reduced ability of *S. oneidensis* to form biofilms (Theunissen et al. 2010). Previous research has also utilized the fusion of redox-sensitive fluorescence protein to BpfA, enabling the visualization of the protein in *S. oneidensis* biofilms under a microscope (Sivakumar et al. 2014).

A recent study reported that the ratio of polysaccharides to proteins in *S. oneidensis* is around 1–2, indicating a higher production of polysaccharides compared to other species (Ding et al. 2014). In previous studies, it has been reported that Pel, Psl, and alginate are the main types of polysaccharides in *Pseudomonas aeruginosa*, with Pel and Psl playing a critical role in the initial attachment stage (Orgad et al. 2011). However, the composition and structure of polysaccharides in *S. oneidensis* during the initial attachment stage have not been fully characterized. It is hypothesized that further studies on polysaccharides are necessary, as they may play an important role in the initial attachment and maturation of biofilms in *S. oneidensis*.

Meanwhile, eDNA has been reported to play an essential role in initial cell attachment and biofilm maturation in *Staphylococcus aureus*. Removal of eDNA from the biofilm matrix significantly weakens biofilm formation (Jermy 2010). The specific function of eDNA in *S. oneidensis* at this stage remains unclear, but it is speculated that eDNA may also contribute to the initial attachment and maturation of biofilms in *S. oneidensis*, similar to other species. Regarding lipid studies, it is proposed that lipids may alter surface charges (Hatzios et al. 2011, Zhao et al. 2011), and surface charges can influence biofilm formation (Ding et al. 2014). Therefore, lipids may also impact biofilm formation in *S. oneidensis*.

### Surface modification: a key strategy to control biofilm formation in *S. oneidensis*

Multiple factors can influence the formation, disruption, or dispersal of biofilms. These factors include surface properties, oxygen and nutrient availability, environmental stress, and metabolites (Tsai et al. 2004, Bazaka et al. 2012). Surface modification is a common approach to enhance or disrupt biofilm formation by altering the surfaces to promote or hinder initial attachment and maturation of cells (Bazaka et al. 2012, Kim et al. 2012, Hou et al. 2013). Previous studies have demonstrated that surface modification can effectively prevent the initial attachment of microorganisms and subsequent biofilm formation (Bazaka et al. 2012). Additionally, research on hollow fiber membranes has shown that surface modification can impact the biofilm formation capability (Hou et al. 2013).

Following medical surgeries, the surfaces of implanted or inserted medical devices are susceptible to biofilm formation, which can lead to health complications (Jass et al. 2003, Shunmugaperumal 2010). Therefore, there is a pressing need for the development of surface materials with antibiofilm properties. By designing improved antibiofilm surface materials, it becomes possible to create medical devices that effectively prevent such infections associated with biofilm formation.

### Nutrient and oxygen: vital factors influencing the biofilm matrix in *S. oneidensis*

In addition to surface properties, the environment is another crucial factor that influences biofilm formation and dispersal. Nutrient availability plays a significant role in shaping the biofilm matrix (Sawyer and Hermanowicz 1998, Hunt et al. 2004, Dominguez-Zacarias et al. 2006). Cells and biofilms require nutrients for growth, and as the nutrient supply becomes depleted, biofilms initiate detachment, with cells dispersing into the environment in search of new surfaces for attachment (Hunt et al. 2004, Dominguez-Zacarias et al. 2006).

Similarly, oxygen availability affects the growth and development of aerobic cells and biofilms. Research has shown that oxygen can promote biofilm formation in *Shewanella putrefaciens* CN32 through the involvement of diguanylate cyclase and an adhesin (Wu et al. 2013). In matured *S. oneidensis* biofilms, when the flow of medium is stopped for several minutes, the biofilms initiate dispersal due to oxygen depletion (Thormann et al. 2005). Thus, nutrient and oxygen availability are important factors that influence the biofilm matrix.

Heavy metals are another influential factor that impacts the biofilm matrix. Taking chromium as an example, a previous study reported that hexavalent chromium induces biofilm detachment. In this study, *S. oneidensis* MR-1 biofilm was grown using modified M1 media. Once the biofilms had developed, hexavalent chromium was introduced into the media, resulting in biofilm detachment. The study revealed that while *S. oneidensis* MR-1 biofilm is capable of reducing Cr(VI) to Cr(III), the presence of Cr(VI) induces biofilm detachment.

### Quorum sensing in *S. oneidensis*: unveiling the language of biofilm communication

In scenarios involving nutrient and oxygen limitation, an intriguing research topic arises: how do cells communicate with each other? Within mature biofilms, certain cells may experience insufficient nutrient or oxygen supply, typically in the inner regions due to limited nutrient dispersion, while other cells, predominantly in the outer regions, continue to receive ample nutrient and oxygen. In such cases, starved cells must communicate their inability to survive and their intention to disperse. Therefore, there is a necessity for cell-to-cell communication. Researchers have discovered that cells employ a quorum sensing system for communication (Flickinger et al. 2011). Quorum sensing facilitates biofilm formation, enabling bacteria to persist for longer periods, and is regulated by c-di-GMP, a second messenger that plays a crucial role in quorum sensing and subsequent biofilm formation (Sharma et al. 2014). Often, high concentrations of c-di-GMP lead to increased biofilm formation capability while reducing cellular motility (Ueda and Wood 2009, Sharma et al. 2014). Quorum sensing is significant as it serves as the communication language among cells. Understanding this language allows scientists to manipulate biofilms, promoting the growth of more robust biofilms or inducing dispersal, depending on the specific context.

### Polyamines in biofilm dynamics: balancing formation and dispersal in *S. oneidensis*

Self-produced metabolites serve as another influential factor that can impact biofilm formation. Various types of self-produced metabolites have been identified to affect the stability of biofilms. Polyamines, such as putrescine, cadaverine, spermidine, and spermine, are organic compounds with two or more primary amino groups. Although the synthesis pathways of polyamines in cells are well-regulated, their exact functions within cells are still not completely understood (Rato et al. 2011).

Numerous studies have reported that polyamines are essential for the growth of cells and biofilms (Patel et al. 2006, Wortham et al. 2010, Sakamoto et al. 2012, Karatan and Michael 2013). For instance, norspermidine has been suggested to enhance biofilm formation in Vibrio cholerae, while spermidine reduces biofilm formation (Karatan et al. 2005, McGinnis et al. 2009). In *Bacillus subtilis*, D-amino acids and norspermidine induce biofilm dispersal (Xu and Liu 2011, Kolodkin-Gal et al. 2012), although some studies have raised questions regarding these findings (Hobley et al. 2014). Overall, the precise role of polyamines in cell metabolism and biofilm formation remains incompletely understood. However, it is evident that polyamines can influence cell growth and the composition of the biofilm matrix. It is hypothesized that under normal conditions, polyamines may enhance biofilm formation. Nevertheless, at high concentrations, polyamines may affect biofilm stability by altering the interactions between matrix components, potentially leading to biofilm dispersal. Therefore, self-produced metabolites represent another significant factor that can influence biofilm formation.

### From motility to metal reduction: unraveling the biofilm phenomenon in *S. oneidensis*

*Shewanella oneidensis* MR-1 is an environmental bacterium that was first isolated in 1988 (Myers and Nealson 1988). It is a nonpathogenic bacterium known for its ability to reduce metals. *Shewanella oneidensis* cells exhibit various modes of motility, including swimming, swarming, and twitching, and the maturation process of its biofilms typically takes ~96 h, with a continuous supply of nutrients. Mature *S. oneidensis* biofilms form three-dimensional structures with a thickness ranging from 20 to 50 µm. The biofilm matrix of *S. oneidensis* primarily consists of proteins, polysaccharides, and eDNA. Notably, the polysaccharide content in *S. oneidensis* biofilm matrix is comparatively high compared to that of *P. aeruginosa*, with a protein-to-polysaccharide ratio of ~1:1. Additionally, the cohesiveness and robustness of *S. oneidensis* biofilms can be enhanced through genetic manipulation (Ding et al. 2014).

*Shewanella oneidensis* is capable of surviving and forming biofilms under both aerobic and anaerobic conditions. In aerobic conditions, the cells utilize oxygen as an electron acceptor. Under anaerobic conditions, *S. oneidensis* can respire using various electron acceptors, including metal ions, metal oxides, and solid electrodes. It demonstrates the ability to reduce different types of metals, such as manganese (Myers and Nealson 1988), chromium (Ding et al. 2014), iron (Ahmed et al. 2012) and palladium (Ng et al. 2013a). This metal-reducing capability of the *S. oneidensis* biofilm matrix holds significant potential for environmental applications.

The ingestion of heavy metals poses a significant risk to human health (Tchounwou et al. 2012). Hexavalent chromium, for instance, is a particularly concerning heavy metal. In many developing countries, the direct disposal of hexavalent chromium into waterways without proper pretreatment is a common practice in industrial production. Consequently, individuals who consume water contaminated with high concentrations of hexavalent chromium may experience stomach ulcers and an increased risk of stomach cancer. Consequently, the development of technologies to remove heavy metals from water sources is crucial.

Various methods have been employed for the removal of heavy metals, including those based on physical adsorption (Yavuz et al. 2006, Di Natale et al. 2007) or chemical reactions (Lv et al. 2014, Mystrioti et al. 2014). However, these methods have certain limitations. Physical adsorption methods may not prevent the desorption of chromium over long timescales (Bai and Abraham 2003, Gupta and Rastogi 2008), and chemical methods relying on oxidation–reduction processes can be expensive due to the required chemical materials. In this context, research on novel bioremediation approaches shows promise.

*Shewanella oneidensis* MR-1, a metal-reducing bacterium, offers an emerging bioremediation technique through its biofilm matrix. This technique demonstrates high efficiency while having minimal negative environmental impacts. Previous studies have utilized genetic manipulation to construct more robust and cohesive biofilms, thereby improving the efficiency of chromium reduction. By harnessing the metal-reducing capabilities of *S. oneidensis* biofilms, this research holds potential for effective and environmentally friendly heavy metal remediation.

Uranium (U) is a naturally radioactive element that undergoes a series of alpha or beta particle emissions, ultimately transforming into the stable element lead. While uranium has applications in electricity generation, nuclear power, and military weaponry, industrial activities have also led to environmental contamination (Davesne and Blanchardon 2014). Uranium contamination in sites managed by the U.S. Department of Energy (DOE) poses significant challenges and incurs substantial remediation costs due to its presence in soils and groundwater (Ahmed et al. 2012). One such site is Hanford 300A, characterized by heavy uranium contamination in soils, sediments, and groundwater (Sivaswamy et al. 2011).

Researchers have explored the potential of *S. oneidensis* sp. HRCR-1 in immobilizing uranium within biofilm matrices. In a study conducted at a hollow fiber membrane biofilm reactor, scientists isolated loosely associated EPS and bound EPS from *S. oneidensis* sp. HRCR-1 biofilms and assessed their reactivity with U(VI). The findings revealed that the isolated cell-free EPS fractions, upon reduction, were able to reduce U(VI) as well. Polysaccharides present in the EPS likely contributed to U(VI) sorption, predominantly in the loosely associated EPS, while redox-active components, particularly within the bound EPS, potentially facilitated U(VI) reduction (Cao et al. 2011). These results highlight the potential of the biofilm matrix of *S. oneidensis* sp. HRCR-1 in immobilizing uranium, offering insights for remediation strategies in uranium-contaminated environments.

In the realm of industrial production, nanomaterials have emerged as key components. Efficient production methods for nanomaterials have become an intriguing area of research. Previous studies have explored the utilization of *S. oneidensis* MR-1 biofilms to produce palladium and silver nanoparticles through reduction processes (Ng et al. 2013a, b). or instance, the reduction of tetrachloropalladate (Pd(II)) by the dissimilatory metal-reducing capabilities of *S. oneidensis* MR-1 led to the production of catalytic palladium (Pd) nanoparticles, offering a novel approach in nanomaterial production (Ng et al. 2013b).

Another study investigated the use of *S. oneidensis* MR-1 for the production of extracellular silver nanoparticles. The research highlighted the significant roles played by outer membrane c-type cytochromes MtrC and OmcA in the synthesis of these nanoparticles. The absence of c-type cytochromes on the cell surface of *S. oneidensis* resulted in a reduction in the particle size of the extracellular biogenic silver nanoparticles. This finding suggests the possibility of controlling the size and activity of such nanoparticles through the regulated expression of genes encoding surface proteins (Ng et al. 2013a).

These studies showcase the potential of *S. oneidensis* MR-1 biofilms as a platform for efficient production of nanomaterials, demonstrating the ability to generate catalytic palladium nanoparticles and control the size of extracellular silver nanoparticles. These findings open up exciting possibilities for the development of sustainable and controlled nanomaterial production processes.

### Biochemical pathways of heavy metal reduction in *S. oneidensis* MR-1: insights into electron transfer mechanisms

In 1988, *S. oneidensis* was initially isolated from the environment, and it was discovered to have the ability to reduce manganese (Myers and Nealson 1988). Subsequent research has revealed that this species can also reduce various other metals, including iron (Caccavo et al. 1997), uranium (Cao et al. 2011), palladium (Ng et al. 2013b), and chromium (Ding et al. 2014). In aerobic environments, *S. oneidensis* prefers oxygen as the electron acceptor, while in anaerobic environments, it utilizes metals as electron acceptors for metabolism. Several carbonate hydrates, such as lactate (Ng et al. 2013a) and pyruvate (Pinchuk et al. 2011), can serve as electron donors and carbon sources. However, the growth of *S. oneidensis* is not observed when glucose is added, suggesting that glucose may not function as an electron donor or carbon source for this species.

**Figure 2.**
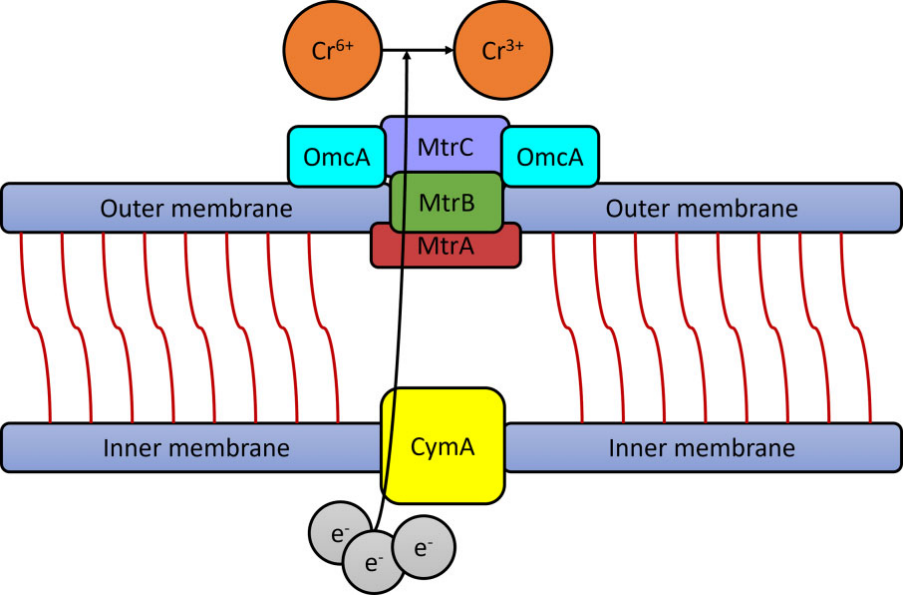
The function of c-type cytochromes MtrC and OmcA in *S. oneidensis* electron transfer. The theory is derived from previous studies (Belchik et al. 2011, Richardson et al. 2012), and the figure has been drawn by the authors of this paper.

The reduction of heavy metals by *S. oneidensis* requires electron transfer. The mechanisms of electron transfer have been explained through different hypotheses, including involvement of cytochromes MtrC and OmcA (Belchik et al. 2011, Richardson et al. 2012), electron shuttles (Wu et al. 2013), and nanowires (Gorby et al. 2006, El-Naggar et al. 2010, Pirbadian et al. 2014). In *S. oneidensis* MR-1, electron transfer occurs through cytochromes. MtrC and OmcA are outer membrane c-type cytochromes in *S. oneidensis* MR-1. Previous studies have reported that *S. oneidensis* cells utilize cytochromes MtrC and OmcA for respiration on hematite (Mitchell et al. 2012) and the reduction of extracellular hexavalent chromium (Belchik et al. 2011). In microbial fuel cells, cytochromes MtrC and OmcA have also been found to play a crucial role in transferring electrons from *S. oneidensis* to oxide electrodes (Jani et al. 2010). The electron transfer system involving cytochromes is illustrated in Fig. 2. MtrA and MtrB are membrane proteins, while c-type cytochromes MtrC and OmcA attach to the outer membrane, forming an electron transfer pathway at the cell membrane. Another study focusing on *S. putrefaciens* MR-1 discussed the presence of the CymA protein in the cytoplasmic membrane and soluble fraction. This protein shares partial amino acid sequence homology with multiheme c-type cytochromes found in other bacteria, which are involved in the transfer of electrons from the cytoplasmic membrane to acceptors in the periplasm. The electrons in *S. oneidensis* cells can pass through the pathway (formed by CymA, MtrA, MtrB, MtrC, and OmcA) to the exterior, initiating reduction reactions. The heavy metals then accept the electrons and undergo reduction.

An alternative explanation for electron transfer in *S. oneidensis* MR-1 involves the role of electron shuttles. It has been found that *S. oneidensis* MR-1 can secrete flavins as electron shuttles, which play a crucial role in extracellular electron transfer. The study reports that the secretion of flavins and subsequent microbial extracellular electron transfer can be significantly influenced by electron acceptors (Wu et al. 2013). The presence of electron shuttles for extracellular electron transfer has also been observed in other *Shewanella* species (Turick et al. 2002, Wu et al. 2014, Zhu et al. 2014).

In recent years, a new theory called “nanowires” has emerged to explain electron transfer in *Shewanella* species (Gorby et al. 2006, El-Naggar et al. 2010, Pirbadian et al. 2014). Researchers have reported that nanowires are extensions of the extracellular electron transport components in the outer membrane and periplasmic space (Pirbadian et al. 2014). Another study demonstrated that nanowires in *S. oneidensis* MR-1 exhibit electrical conductivity over micrometer-length scales, with electron transport rates up to 10^9^/s at 100 mV and a measured resistivity on the order of 1 Ω cm (El-Naggar, Wanger et al. 2010).

## Conclusion

Biofilms represent a fascinating and widespread mode of microbial life, with their distinctive EPS providing a protective and adaptive environment for microorganisms. EPS contribute to the physiological differences between biofilm cells and their planktonic counterparts, enabling survival in hostile conditions and facilitating dispersion for colonization of new habitats. In this review, we have explored the fundamental aspects of biofilm matrix, including its life cycle, matrix components, and factors influencing formation and stability. Taking *S. oneidensis* as a model organism, we have specifically examined its biofilm matrix. *Shewanella oneidensis*, as an environmentally isolated and nonpathogenic bacterium, exhibits remarkable capabilities for heavy metal reduction and immobilization. The unique characteristics of *S. oneidensis* and its biofilm matrix offer promising prospects for various bioapplications, particularly in the areas of heavy metal remediation and immobilization.
